# Combination of cetuximab with chemoradiation, trastuzumab or MAPK inhibitors: mechanisms of sensitisation of cervical cancer cells

**DOI:** 10.1038/sj.bjc.6605216

**Published:** 2009-08-04

**Authors:** D D Meira, V H de Almeida, J S Mororó, I Nóbrega, L Bardella, R L A Silva, R M Albano, C G Ferreira

**Affiliations:** 1 Division of Clinical Research, Research Coordination, Instituto Nacional de Câncer (INCA), Rio de Janeiro, Rua André Cavalcanti, Brazil; 2 Departamento de Bioquímica, Universidade do Estado do Rio de Janeiro, Rio de Janeiro, Brazil

**Keywords:** cetuximab, trastuzumab, PD153035, PD98059, chemoradiation, cervical cancer

## Abstract

**Background:**

Cervical cancer (CC) annually kills 288 000 women worldwide. Unfortunately, responses to chemoradiation are partial and are of short duration. As anti-EGFR monoclonal antibodies sensitise tumours, we investigated cetuximab's toxicity plus chemoradiation on CC cells, which express different EGFR levels.

**Methods:**

EGFR, HER2, AKT and MAPK expression and phosphorylation status were determined by western blotting. Cytotoxicity was assessed by MTT or clonogenic assays (CA) in cell lines treated with cetuximab alone or in combinations.

**Results:**

Cetuximab with cisplatin and radiation achieved maximum cytotoxic effects for A431, Caski and C33A cells (high, intermediate and low EGFR expression, respectively) in CA. Cetuximab efficiently decreased MAPK and AKT phosphorylation in A431 cells but slightly less in Caski and C33A cells. To check whether further EGFR, HER2 or MAPK inhibition would improve cetuximab's cytotoxicity, we combined it with an EGFR tyrosine kinase inhibitor (TKI), trastuzumab or a MEK1/2 inhibitor (PD98059). In Caski, but not in C33A cells, cetuximab cooperated with the TKI, reducing cell survival and AKT and MAPK phosphorylation. However, cetuximab with trastuzumab or PD98059 reduced survival and MAPK phosphorylation of both cell lines.

**Conclusion:**

Our data suggest that cetuximab combined with chemoradiation, trastuzumab or MAPK inhibitors has useful applications for CC treatment, independently of EGFR expression.

Cervical cancer (CC) is a common malignancy which kills 288 000 women annually. Radical hysterectomy and radiation therapy are standard treatments for early-stage invasive CC, whereas pelvic radiation has been used for locally advanced cancers. Recently, an improved survival of patients with locally advanced CC was reported when platinum-based chemotherapy was combined with radiation treatment (Ota *et al*, 2007). However, despite technological advances, up to 35% of all CC patients will develop persistent/recurrent/metastatic disease (Bellone *et al*, 2007).

Infection of human keratinocytes by oncogenic HPV subtypes is critical for cervical carcinogenesis, however, it is not sufficient for cancer development. Among other molecular cofactors investigated, epidermal growth factor (EGF) receptor (EGFR) overexpression is frequent in CC and is an independent predictor of poor prognosis in advanced stage tumours (Kersemaekers *et al*, 1999). Epidermal growth factor receptor, a 170-kDa transmembrane glycoprotein, belongs to the ErbB/HER family of receptors, which include HER2 (ErbB2), HER3 (ErbB3) and HER4 (ErbB4). Ligand binding leads to the formation of homo- or heterodimers with other members of the family, receptor autophosphorylation and activation of signalling pathways, which regulate cell proliferation, survival and transformation (Citri and Yarden, 2006). A major EGFR signalling route, the mitogen-activated protein kinases (MAPK) pathway is often deregulated in cancer cells. The activation of this pathway, comprised by Ras, Raf, MEK1/2 and extracellular signal-regulated kinase (ERK)1/2, leads to tumour development and progression through the activation of transcription factors that stimulate cell proliferation and metastasis (Dhillon *et al*, 2007; Katz *et al*, 2007).

Anti-EGFR monoclonal antibodies (MAbs) are important molecular-targeted drugs for cancer treatment. Among them, cetuximab (Erbitux), a recombinant chimeric human-murine MAb, is one of the most promising and clinically effective (Mendelsohn and Baselga, 2000). It binds EGFR with higher affinity than the original ligands, preventing receptor activation, inducing EGFR internalisation and degradation, inhibiting cell proliferation and angiogenesis and promoting antibody-dependent cellular cytotoxicity (ADCC; Mendelsohn and Baselga, 2000; Vincenzi *et al*, 2008). Cetuximab induces the radiosensitisation of different cell types both *in vitro* and *in vivo*, including head and neck, lung, colon, ovarian and breast cancer cell lines (Vincenzi *et al*, 2008). Another important member of the ErbB family is HER2, which is often overexpressed in breast, ovarian, colon and gastric cancer. The recombinant humanised anti-HER2 trastuzumab (Herceptin), has high affinity for HER2 and induces its downregulation, with inhibition of proliferation of breast cancer cell lines *in vitro* and *in vivo* (Mendelsohn and Baselga, 2000; Vincenzi *et al*, 2008).

As far as we know, data on the combination of cetuximab plus chemoradiation in CC are lacking; thus, in this study, we determined cetuximab's activity either alone or combined with cisplatin and/or radiotherapy towards CC cell lines which express different levels of EGFR. We observed that an efficient inhibition of the MAPK pathway is a determinant factor for sensitisation to cetuximab treatment. Moreover, our data suggest that cetuximab, given together with chemoradiation, trastuzumab or MAPK inhibitors, could have important applications to treat CC, independently of EGFR expression status.

## Materials and methods

### Cell lines

A431 cell line was kindly provided by Dr Giuseppe Giaccone (University Hospital Vrije Universiteit, The Netherlands). Caski and C33A cells were provided by Dr Luisa L Villa (Ludwig Institute for Cancer Research, Brazil).

### Chemicals

Cetuximab was generously provided by Merck KGaA (Darmstadt, Germany). PD98059 and PD153035 were obtained from Calbiochem (Nottingham, UK). Trastuzumab was provided by Instituto Nacional de Câncer (Rio de Janeiro, Brazil).

### MTT and clonogenic assays

For MTT, cells were incubated with cetuximab or cisplatin at different concentrations or cetuximab in the presence/absence of PD98059 (25 *μ*M), a MEK1/2 inhibitor (Friday and Adjei, 2008). After 72 h, cells were incubated with MTT reagent (Sigma, St Louis, MO, USA) and processed as previously described (Meira *et al*, 2005). Concentrations resulting in cell growth inhibition of 30 (IC_30_), 50 (IC_50_) and 80% (IC_80_) were calculated for cisplatin. For other treatments, cell viability was expressed as a percentual of controls (%CT).

For clonogenic assays (CA), cells were either left untreated or irradiated with a ^60^Co-THERATRON-780C irradiator (Theratronics, Canada) and allowed to grow for 14 days. For the evaluation of cetuximab alone, or with trastuzumab, or with PD153035, a specific EGFR TKI, cells were incubated with cetuximab (100 *μ*g ml^−1^) and trastuzumab (10 *μ*g ml^−1^), or with PD153035 (0.1 *μ*M) for 72 h. For the combination experiments with chemoradiation, cetuximab (100 *μ*g ml^−1^) and cisplatin (at different concentrations) were added, and 6 h later, cells were irradiated and maintained at 37°C for 72 h. Cells were allowed to proliferate in fresh medium for 10 days and the number of colony-forming units stained with crystal violet was expressed as the surviving fraction (SF).

### Drug interaction analysis

The cytotoxic effects of the combination of cetuximab and PD98059 or PD153035 or trastuzumab were analysed according to Fischel *et al* (2005), in which *R* means ratio and if *R*<0.8, the association is considered to be synergistic, 0.8 <*R*<1.2, additive and *R*>1.2, antagonistic.

### Cell-cycle analysis

Cells were incubated alone or in the presence of cetuximab (100 *μ*g ml^−1^), as previously described (Janmaat *et al*, 2003). After 24 h, cell-cycle phase distribution was analysed by flow cytometry using propidium iodide (PI) staining and the resulting DNA content were analysed on a Becton Dickinson FACScalibur using ModFitLT V2.0 software (Becton Dickinson, CA, USA).

### Hoechst staining

Cells monolayers were treated with cetuximab (100 *μ*g ml^−1^) for 24 h and DNA was stained with Hoechst 33342 (Sigma). Morphological signs of apoptosis were analysed in duplicate by fluorescence microscopy and each experiment was repeated at least three times.

### Western blotting analysis

Cells were incubated for 4 h in the presence of cetuximab (100 *μ*g ml^−1^) alone or followed by a 15-min incubation with EGF (10 ng ml^−1^) as previously described (Meira *et al*, 2009). For combination experiments, cells were treated as described above, plus 4 h of incubation with trastuzumab (10 *μ*g ml^−1^), or 1 h of incubation with PD98059 (25 *μ*M) or PD153035 (0.1 *μ*M) alone or combined with cetuximab before the incubation with EGF. As C33A cells express low levels of EGFR, more protein was loaded (80 *μ*g, as opposed to 50 *μ*g for the other cells) on the gels to observe the phosphorylation status of the receptor. Primary antibodies against total and phosphorylated EGFR, HER2, AKT and MAPK (all from Cell Signaling Technology, Beverly, MA, USA) were used. Immunoblots were detected using the enhanced chemiluminescence (ECL) reagent (GE Health Care, Saˇo Paulo Brazil) and bands were quantified with Labworks, version 4.6 (Bio-Rad, USA).

### *EGFR* mRNA expression by real-time RT-PCR

Total RNA was isolated from cell lines and used for reverse transcription (RT). Real-time RT-PCR was performed with the QuantiTect SYBR Green PCR Master Mix (Qiagen, Valencia, CA, USA) and the relative expression level of *EGFR* mRNA was calculated using the comparative *C*_T_ method (ΔΔ*C*_T_) as described elsewhere (Livak and Schmittgen, 2001).

### EGFR cell surface expression by flow cytometry

As previously described (Meira *et al*, 2009), cells were incubated either with a murine anti-EGFR Mab (0.1 *μ*g *μ*l^−1^; BD Pharmingen, San Diego, CA, USA) or cetuximab (0.1 *μ*g *μ*l^−1^) for 1 h on ice. After washing, secondary antibodies (Caltag Laboratories, Burlingame, CA, USA) were added and samples were analysed on a FACScalibur using CELLQuest software (Becton Dickinson, San Jose, CA, USA).

### *In vitro* ADCC assay

Antibody-dependent cellular cytotoxicity assay was performed with the kit CytoTox 96 Non-Radioactive Cytotoxicity Assay (Promega, Madison, WI, USA). Cells were incubated alone or in the presence of 4 *μ*g ml^−1^ of cetuximab for 4 h and exposed to peripheral blood mononuclear cells (PBMC) at effector/target ratios (E/T) of 20 : 1 for 4 h and specific cytolysis (ADCC) was measured as previously described (Meira *et al*, 2009).

### Determination of VEGF secretion

Cells were incubated alone or in the presence of cetuximab (100 *μ*g ml^−1^) for 24 or 48 h. VEGF protein concentration in the culture medium was determined using human VEGF ELISA Development Kit (Peprotech Inc., Rock Hill, NJ, USA).

### Statistical analysis

All experiments were done in triplicate and the values represent an average of at least three independent experiments. Statistical analyses were performed using GraphPad Prism 3.0 (GraphPad Software Incorporated, CA, USA). Quantitative experiments were analysed by Student's *t*-test. One-way analysis of variance (ANOVA) with Tukey's post test was used to analyse the combination of cetuximab, cisplatin and RxT *vs* double or individual treatments by CA. All *P* values resulted from the use of two-sided tests and were considered significant when <0.05.

## Results

### Differential effects of RxT, cisplatin and cetuximab on cell proliferation and cell-cycle kinetics

**Figure 1:**
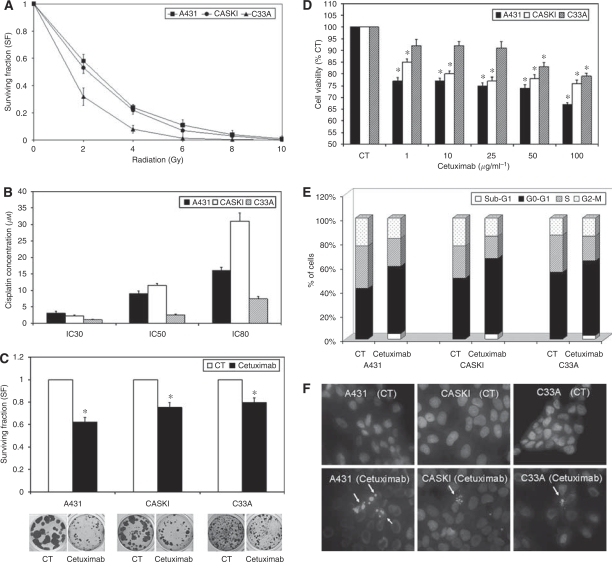
Effect of RxT, cisplatin or cetuximab treatments on the proliferation and cell-cycle distribution of A431, Caski and C33A cells. (**A**) Effects of different doses of RxT in clonogenic assays (CA). (**B**) Effects of different concentrations of cisplatin in MTT assay. (**C**) Effects of cetuximab (100 *μ*g ml^−1^) in CA. (**D**) Effects of cetuximab treatment in MTT assay. (**E**) Effects of cetuximab treatment (100 *μ*g ml^−1^) on cell-cycle phase distribution analysis by flow cytometry. (**F**) Induction of apoptotic bodies by cetuximab treatment (100 *μ*g ml^−1^) visualised by Hoechst staining. Student's *t*-test ^*^P<0.05, compared to controls.

We examined the antiproliferative effects of isolated RxT, cisplatin and cetuximab treatments on A431, Caski and C33A cells (Figures 1A–F), which express high to low EGFR levels, respectively (see Figures 3A and 5A). A431 growth is impaired by EGFR inhibitors (Janmaat *et al*, 2003), so we used this cell line for comparison with the CC cell lines. A431 and Caski cells showed similar resistance to RxT and low cisplatin concentrations (IC_30_) in CA and MTT assays (Figures 1A and B), but Caski cells showed increased resistance at higher cisplatin concentrations (IC_50_ and IC_80_; Figure 1B). C33A cells were, in turn, quite sensitive to both treatments (Figures 1A and B). Cetuximab treatment decreased the viability of all cell lines in CA and MTT experiments at all concentrations tested (Figures 1C and D). These effects were related to an increase of 14.1, 12.6 and 6.5% in cell number at G_0_/G_1_ for A431, Caski and C33A cells on cetuximab treatment, respectively (Figure 1E) and a decrease in the population of cells at S and G_2_/M phases when compared to controls (CT; Figure 1E).

Apoptotic cells are usually found in the sub-G_1_ phases and, accordingly, 24-h cetuximab treatment increased this cell population by 4.6, 3.9 and 2.9% in A431, Caski and C33A cells, respectively (Figure 1E). Additionally, apoptotic cells were also observed by Hoechst staining in all cell lines after cetuximab treatment (Figure 1F) and, even in C33A cells, which express low EGFR levels, a reduction of 21% in cell proliferation was obtained, especially at high MAb concentration (100 *μ*g ml^−1^; Figure 1D).

### Cetuximab induces chemo/radiosensitisation of CC cells

**Figure 2:**
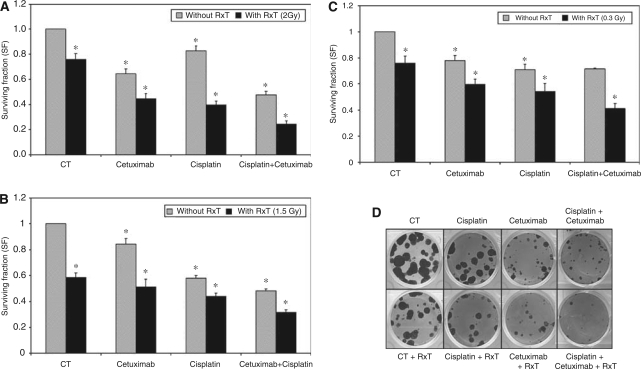
Effects of cetuximab (100 *μ*g ml^−1^) alone or combined with cisplatin (1.0, 0.5 and 0.15 *μ*M for A431, Caski and C33A cells, respectively) and/or RxT (2.0, 1.5 and 0.3 Gy for A431, Caski and C33A cells, respectively) on survival in CA. (**A**) A431, (**B**) Caski and (**C**) C33A cells. (**D**) Representative picture of A431 cells under different treatments as described in material and methods. One-way analysis of variance (ANOVA) with Tukey's post test ^*^P<0.05, when compared to control cells.

We evaluated whether the combination of cetuximab (100 *μ*g ml^−1^) with RxT and/or cisplatin could enhance the cytotoxic effects observed in CA. Isolated cetuximab, cisplatin and RxT treatments decreased the survival of all cell lines (Figures 2A–D) but the combination of cetuximab with either RxT or cisplatin enhanced these effects (*P*<0.05). Furthermore, the triple combination of cetuximab, cisplatin and RxT achieved maximum effects for all cell lines in CA (demonstrative pictures of A431 cells are shown in Figure 2D). Caski cells are HPV-positive and express intermediate levels of EGFR and HER2, resembling typical CC tumours. In this cell line, the triple combination reached up to 70% inhibition in CA (Figure 2B) whereas in C33A cells, an inhibition of 60% was observed (Figure 2C).

### The CC cell lines express different basal levels of total and phosphorylated EGFR, HER-2, AKT and ERK1/2

**Figure 3:**
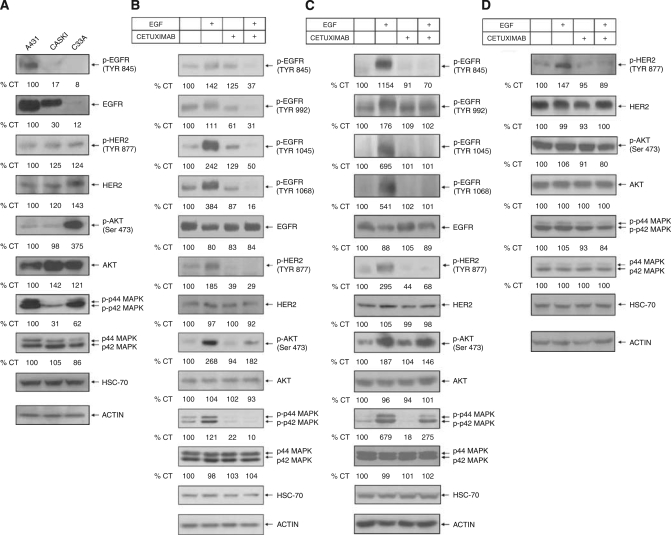
Western blotting analysis of basal and phosphorylated signalling pathways before and after cetuximab (100 *μ*g ml^−1^) treatment. (**A**) Basal levels of total and phosphorylated EGFR, HER2/*neu* and downstream signalling proteins. (**B**, **C** and **D**) Effects of cetuximab on EGF-induced activation of EGFR (Tyr 845, 992, 1045 and 1068), HER-2/*neu*, AKT and ERK1/2 of A431, Caski and C33A cells, respectively, detected by western blotting.

A431 cells strongly express EGFR (Janmaat *et al*, 2003) whereas Caski and C33A cells show moderate and low expression levels, respectively (Figure 3A). Additionally, we confirmed EGFR expression by real-time RT-PCR and by FACS analysis. The relative *EGFR* mRNA level of A431 is high and Caski cells express two times more *EGFR* than C33A (Figure 5A). Moreover, FACS analysis showed that both a murine anti-EGFR MAb and cetuximab could detect high EGFR expression on the surface of A431 cells and intermediate and low levels in Caski and C33A cells, respectively (Figure 5B). HER2 expression was more homogenous among the cell lines with Caski and C33A cells expressing 20 and 40% more HER2 than A431 cells, respectively. The basal phosphorylation status of EGFR and HER2 was inversely correlated, with higher levels of p-EGFR in A431 cells and higher levels of p-HER2 in C33A and Caski cells (Figure 3A).

On EGF binding, the major signalling pathways activated are the MAPK and AKT cascades (Citri and Yarden, 2006). A431 and Caski cells show low basal levels of p-AKT whereas C33A cells have a much higher level. A431 and C33A cells had higher levels of activated ERK1/2 (p-p44/42 MAPK) but no significant differences in total MAPK were observed among the cell lines (Figure 3A).

### Cetuximab inhibits EGFR and HER2 phosphorylation

To investigate the molecular determinants for cetuximab's effects in MTT and CA, we analysed EGFR phosphorylation by WB in cells treated with cetuximab (100 *μ*g ml^−1^) alone or in the presence of EGF. Receptor phosphorylation was increased by EGF in A431 and Caski cells, whereas cetuximab reduced it (Figures 3B and C). Epidermal growth factor and cetuximab also induced a slight decrease in the total amount of EGFR in these cells (Figures 3B and C).

Epidermal growth factor receptor can interact with another member of the ErbB family, HER2, to form heterodimers that are very potent in activating signal transduction pathways (Citri and Yarden, 2006). On cetuximab treatment there were no changes in total HER2 in the CC cell lines, whereas EGF-induced HER2 phosphorylation was inhibited in A431 and Caski cells (Figures 3B and C). Interestingly, in C33A cells, which express more HER2 than EGFR (Fig 3A), cetuximab markedly reduced EGF-induced HER2 phosphorylation (Figure 3D).

There were no changes in total AKT and MAPK proteins in all cell lines on cetuximab treatment (Figures 3B–D). Epidermal growth factor increased AKT and ERK1/2 phosphorylation in A431 and Caski cells but in C33A cells there was only a slight increase. This was not unexpected, because both pathways are activated in this cell line (Figure 3A). Cetuximab inhibited EGF-induced AKT phosphorylation more strongly in A431 cells and less so in Caski and C33A cells. Indeed, it markedly reduced EGF-induced ERK1/2 phosphorylation in A431 cells, but in Caski and C33A cells the reduction was more modest (60 and 20% inhibition, respectively; Figures 3B–D), suggesting that persistent signalling through these pathways led to increased survival of Caski and C33A cells, when compared to A431 cells in the presence of cetuximab.

### Cetuximab combined with trastuzumab synergistically reduces cell proliferation and activation of downstream signalling pathways in CC cells

**Figure 4:**
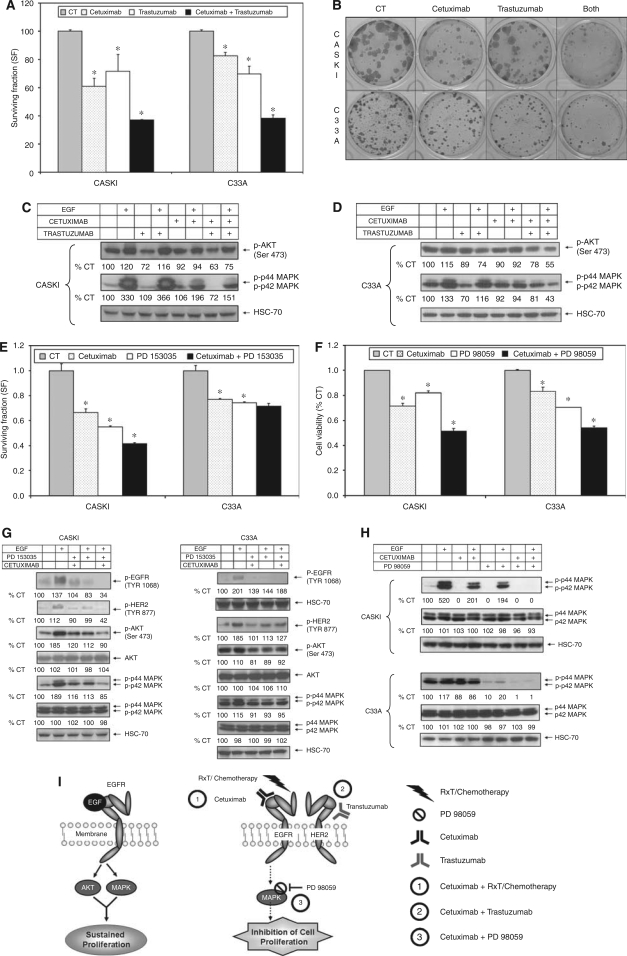

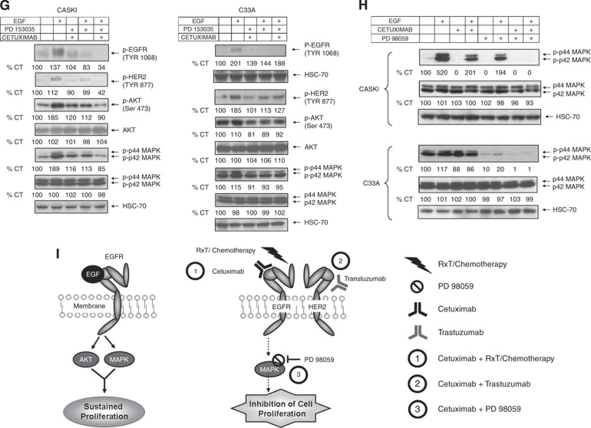
Effects of cetuximab (100 *μ*g ml^−1^) alone or combined with trastuzumab (10 *μ*g ml^−1^), an anti-HER2 MAb, or PD153035 (0.1 *μ*M), an EGFR tyrosine kinase inhibitor, or PD98059 (25 *μ*M), a MAPK pathway inhibitor, on Caski and C33A cells. (**A**) Effects of cetuximab alone or combined to trastuzumab on the survival in CA. (**B**) Representative pictures of Caski and C33A cells under cetuximab and trastuzumab treatments in CA. (**C** and **D**) Western blotting analysis of the inhibition of EGF-induced phosphorylation of AKT and p44/42 (ERK1/2) by cetuximab alone or combined with trastuzumab in Caski and C33A cells, respectively. (**E**) Effects of cetuximab alone or combined with PD153035 on cell survival in CA. (**F**) Effects of cetuximab alone or combined with PD98059 on proliferation by MTT assays. (**G**) Western blotting analysis of the inhibition of EGF-induced phosphorylation of EGFR (Tyr1068), HER-2/*neu*, AKT and p44/42 (ERK1/2) by cetuximab alone or combined with PD153035. To visualise EGFR in C33A cells, 80 *μ*g of protein were loaded on the SDS–PAGE gels as seen by the higher amount of the endogenous control, HSC-70. (**H**) Western blotting analysis of the inhibition of phosphorylation of p44/42 (Erk1/2) by cetuximab and PD98059. Student's *t-*test ^*^P<0.05, when compared to control cells. (**I**) Proposed model of the effects of the combination of cetuximab with chemoradiation or trastuzumab or MAPK pathway inhibitor on CC cell lines. Ligand binding activates signalling through EGFR and triggers the AKT and MAPK pathways. The binding of cetuximab sensitises CC cells to chemoradiation (1) and to trastuzumab (2), leading to cell death independently of EGFR expression levels, but more dependent on EGFR-HER2 signalling. For cells in which activation of the MAPK pathway occurs also through EGFR independent mechanisms, cetuximab inhibition of EGFR sensitises them to PD98059 (3), a MAPK pathway inhibitor, leading to additive effects on the inhibition of cell proliferation.

We speculated that cells expressing higher EGFR/HER2 ratios, such as A431 cells, rely more on EGFR signalling for MAPK pathway activation and cell proliferation, whereas cells with a lower EGFR/HER2 ratio, such as C33A cells, depend more on EGFR/HER2 heterodimer signalling. Based on this assumption, the inhibition of the EGFR/HER2 heterodimer by anti-EGFR (cetuximab) and anti-HER2 (trastuzumab) MAbs should interfere with C33A cell proliferation. As expected, this combination markedly reduced C33A cell colony formation leading to a synergistic interaction (*R*=0.58; Figures 4A and B), with concomitant reduction of MAPK and AKT phosphorylation (Figure 4D). Indeed, an additive effect (*R*=0.84) was also noted for Caski cells, that express intermediate levels of EGFR and HER2 (Figure 3A), with a decrease of almost 60% in cell survival (Figures 4A and B) and inhibition of downstream signalling pathways (MAPK and AKT; Figure 4C). There were no changes in total AKT and MAPK proteins in Caski and C33A cell lines on treatments (data not shown).

### The combination of cetuximab with a TKI inhibits cell proliferation and MAPK phosphorylation in Caski but not in C33A cells

Based on the idea of an EGFR/HER2 heterodimer signalling dependency of C33A cells, we investigated whether further EGFR inhibition with another targeted drug, such as TKIs, affected more Caski than C33A cells. Therefore, we tested the combination of cetuximab with a specific EGFR TKI (PD153035). As expected, combined treatments reduced Caski cell survival leading to an additive interaction (*R*=0.94) when compared to treatments alone (Figure 4E). Additionally, the double treatment in Caski cells was accompanied by a greater reduction of EGFR, HER2, AKT and MAPK phosphorylation (Figure 4G).

Isolated cetuximab or PD153035 treatments reduced the survival of C33A cells in CA by the same proportion, reaching a more modest inhibition of HER2, AKT and MAPK phosphorylation than in Caski cells (Figures 4E and G). In contrast, the combined treatment proved to be antagonistic (*R*=1.28), with no decrease in phosphorylated proteins when compared to either drug alone (Figures 4E and G). Altogether, these data corroborate with our hypothesis that C33A cells are not so dependent on EGFR signalling for proliferation, as double EGFR inhibition with different drugs did not enhance the toxicity achieved by either agent alone. Additionally, the targeting of EGFR and HER2 with two different MAbs showed synergistic inhibitory effects (Figures 4A and B) demonstrating that heterodimer signalling is necessary for C33A cell proliferation. Furthermore, these data indicate that the successful inhibition of the MAPK and/or AKT pathways is a determinant factor for cetuximab efficacy in all CC cell lines.

### Cetuximab combined with PD98059 synergistically reduces cell proliferation and MAPK pathway activation in CC cells

To confirm that the MAPK pathway is relevant for cetuximab response, drug combination experiments with PD98059 (MEK1/2 inhibitor) were performed. Both treatments inhibited Caski and C33A cell proliferation by the same magnitude, with a reduction of 50% and an additive effect for the combination over single-drug treatment in both cell lines (*R*=0.88 and *R*=0.92, respectively; Figure 4F). As expected, Figure 4H shows that a strong inhibition of MAPK phosphorylation was seen in both cell lines on treatment with PD98059 alone or in combination with cetuximab. As the combination of both treatments led to a near complete MAPK cascade blockage, accompanied by a significant reduction of CC cell proliferation, we confirmed that the inhibition of this pathway plays an important role in cetuximab's efficacy.

### Cetuximab induces ADCC in A431 and Caski, but not in C33A cells

**Figure 5:**
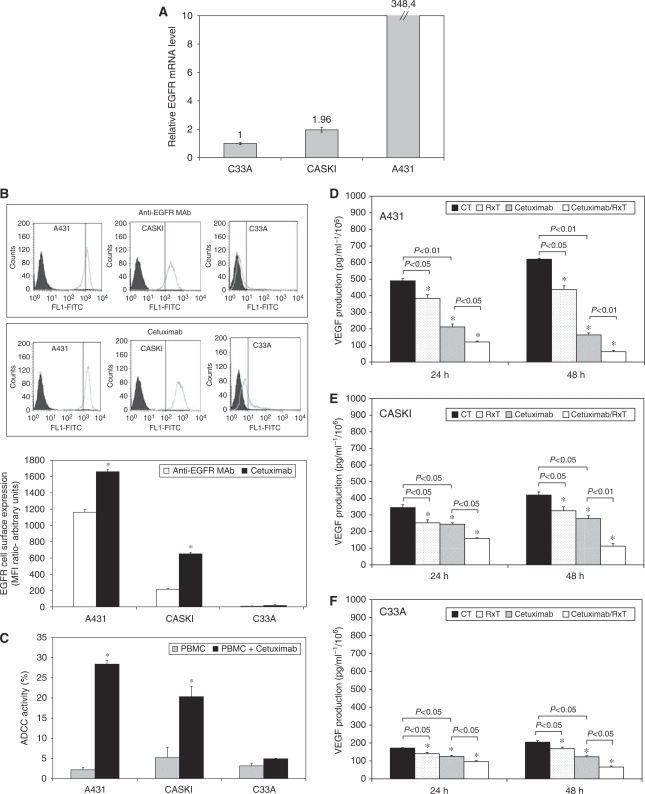
EGFR expression of CC cells, induction of ADCC and modulation of VEGF expression by cetuximab (100 *μ*g ml^−1^) treatment. (**A**) *EGFR* mRNA expression by real-time RT-PCR and (**B**) EGFR cell surface expression measured by flow cytometry. (**C**) ADCC assay. (**D**, **E** and **F**) VEGF protein concentration detected in the culture medium by ELISA in CC cells. Student's *t*-test ^*^P<0.05, when compared to controls.

Antibody-dependent cellular cytotoxicity response is dependent on the number of EGFR molecules per cell and on how efficiently cetuximab recognises its target (Vincenzi *et al*, 2008). FACS analysis showed that cetuximab detected even more cell surface receptors in A431 and Caski cells (*P*<0.05), when compared to a commercially available murine anti-EGFR MAb, although the same was not observed for C33A cells (Figure 5B). Accordingly, at E/T ratios of 20 : 1, cetuximab mediated ADCC in 26.4 and 15.1% of A431 and Caski cells, respectively, but not in C33A cells (1.75%; Figure 5C).

### Cetuximab and RxT cooperate in an additive manner to inhibit VEGF secretion

Anti-EGFR MAbs show suppressive effects on VEGF expression *in vitro* and *in vivo* (Vincenzi *et al*, 2008; Meira *et al*, 2009). To examine whether cetuximab (100 *μ*g ml^−1^) had this effect in CC cells, we tested it alone or combined with RxT (5 Gy) and VEGF expression was analysed by ELISA. Cetuximab or RxT treatments decreased VEGF secretion in all cell lines (Figures 5D–F; *P*<0.05). The combination of cetuximab with RxT for 24 h had an additive effect and, after 48 h of treatment, a further reduction was observed (Figures 5D–F), suggesting that these treatments have the potential of interfering with angiogenesis even in cells that do not express high EGFR levels.

## Discussion

In the past two decades we have seen the successful development of EGFR-targeted drugs, expanding treatment options for cancer patients. Cetuximab is currently being tested in preclinical and clinical studies worldwide to treat several types of cancer. Unfortunately, several issues still remain unaddressed, such as which patients are most likely to have a therapeutic benefit, what are the predictive factors of sensitisation or response to these agents and, most importantly, which are the best strategies for combination with conventional treatments.

Available data suggest that cetuximab enhances chemotherapy and radiotherapy's effects and reverses resistance to some anticancer drugs (Merlano and Occelli, 2007). Cetuximab's blockage of EGFR signalling sensitises several types of cells to RxT (Mendelsohn and Baselga, 2000; Vincenzi *et al*, 2008) and the combination of cetuximab with cisplatin is effective and tolerable to patients (Mendelsohn and Baselga, 2000; Merlano and Occelli, 2007). Our data demonstrated that cetuximab sensitised all tested CC cell lines to RxT and cisplatin treatment, independently of EGFR expression.

Recently, it has been shown that EGFR-negative colon tumours have the potential to respond to cetuximab-based therapies (Chung *et al*, 2005). Moreover, it was demonstrated that HER2 signalling could mediate resistance to TKI in breast cancer cell lines due to the activation of alternative EGFR family receptors (Kong *et al*, 2008). In accordance, EGFR promoted dimerisation and strong activation of HER2 in cells, which acquired cetuximab resistance, with consequent activation of downstream cascades and sustained proliferation (Wheeler *et al*, 2008). These studies suggest that EGFR is responsible for transphosphorylation of HER2 and that high HER2 expression confers increased sensitivity to cetuximab's therapeutic effects. Our data confirmed this hypothesis, because the double inhibition of EGFR and HER2 by cetuximab and trastuzumab further decreased Caski and C33A cell proliferation. Additionally, the response to these drug combinations does not seem to rely solely on EGFR expression levels but also on an effective inhibition of HER family members and of downstream signalling pathways.

In Caski cells, the isolated treatment with cetuximab and with the TKI PD153035 or their combination diminished survival in CA (Figure 4E). However, in C33A cells this combination was antagonic (Figure 4E). This antagonism has also been demonstrated when cetuximab was combined with ZD 1839 (TKI) in head and neck cell lines (Fischel *et al*, 2005). As described for colon, breast and lung cell lines (Mendelsohn and Baselga, 2000; Janmaat *et al*, 2003; Matar *et al*, 2004) the success of the combination of MAbs with TKIs was due to an efficient inhibition of EGFR, HER2, AKT and MAPK phosphorylation, corroborating our observations for Caski cells (Figure 4G). In C33A cells, however, the combination of cetuximab plus PD153035 failed because it did not bring any further reduction in AKT or MAPK activation (Figure 4G).

Recent data described that CC progression was correlated with MAPK pathway activation, showing that this cascade has an important role in CC development (Chen *et al*, 2007; Perez-Plasencia *et al*, 2007). Clinical studies done with MEK inhibitors have examined only single agent effects (Friday and Adjei, 2008) and the combinations with cytotoxic agents are likely to be beneficial for cancer treatment. The importance of the MAPK cascade for CC cell survival was demonstrated when we combined a specific MEK1/2 inhibitor (PD98059) with cetuximab, resulting in an additive inhibition of Caski and C33A cell proliferation. Indeed, based on the basal levels of relevant signalling molecules, we suggest that blockage of p-EGFR, p-HER2 and p-MAPK are predictive factors for cetuximab sensitisation. In accordance, a recent study of head and neck cancer demonstrated that activation of EGFR and HER2 was correlated with MAPK activation and that ERK1/2 inhibition by cetuximab points to these molecules as potential surrogate markers in the clinical setting (Albanell *et al*, 2001). Based on this, we proposed a model of cetuximab's effects in combination with chemoradiation, trastuzumab or MAPK pathway inhibitors on CC cell lines (Figure 4I). Firstly, our data support the idea that combination of cetuximab with chemoradiation is an interesting approach to treat CC, irrespective of EGFR expression. Secondly, this study reported that cetuximab's effects are influenced by HER2 expression and the use of cetuximab in combination with trastuzumab could decrease CC cell survival. Thirdly, we observed that MAPK pathway activation was dominant for CC cell survival and that the blockage of both pathways (EGFR/MAPK) acts synergistically in inhibiting cell proliferation.

Anti-EGFR MAbs can also downregulate VEGF expression (Mendelsohn and Baselga, 2000; Vincenzi *et al*, 2008; Meira *et al*, 2009) and, accordingly, cetuximab alone or with RxT reduced VEGF protein secretion in all cells, indicating the potential role of this combination in the inhibition of angiogenesis *in vivo*. Antibody-dependent cellular cytotoxicity activity is another important anticancer mechanism induced by cetuximab in various cell lines (Bellone *et al*, 2007; Meira *et al*, 2009) and this MAb effectively induced ADCC in A431 and Caski cells, whereas no ADCC was observed in the C33A cell line. Based on these findings we conclude that cell surface EGFR expression is very relevant for cetuximab induction of ADCC, but not as much for inhibiting VEGF secretion and cell proliferation, demonstrating that cetuximab can modulate multiple crucial pathways of CC cell lines in different ways.

In summary, our data suggested that the combination of cetuximab with cisplatin/ RxT, trastuzumab or with MAPK inhibitors could be useful for CC treatment, independently of EGFR expression status. Our preclinical data are encouraging and we hope that these results can be translated into the clinical setting. We believe that appropriately designed clinical trials are required to define the optimum doses and sequence of treatments, and will be interesting to first try these combinations in animal models. Indeed, the understanding of the relative contribution of individual members of the ErbB receptor family and activated downstream pathways in cervical cancer cell proliferation is far from complete, and further validation of our results in experiments involving primary cervical tumours *in vitro* are desired.
